# Supplementary material for: The Construction and Exploration of a Comprehensive MicroRNA Centered Regulatory Network in Foxtail Millet (Setaria italica L.)
Source: Front Plant Sci. 2022 May 6;13:848474. doi: 10.3389/fpls.2022.848474 (PMC9121102; doi:10.3389/fpls.2022.848474)
Supplement: Supplementary file 1 [file Data_Sheet_1.zip › Sit-miRNovel8_Seita.4G037500.1_391_TPlot.pdf]

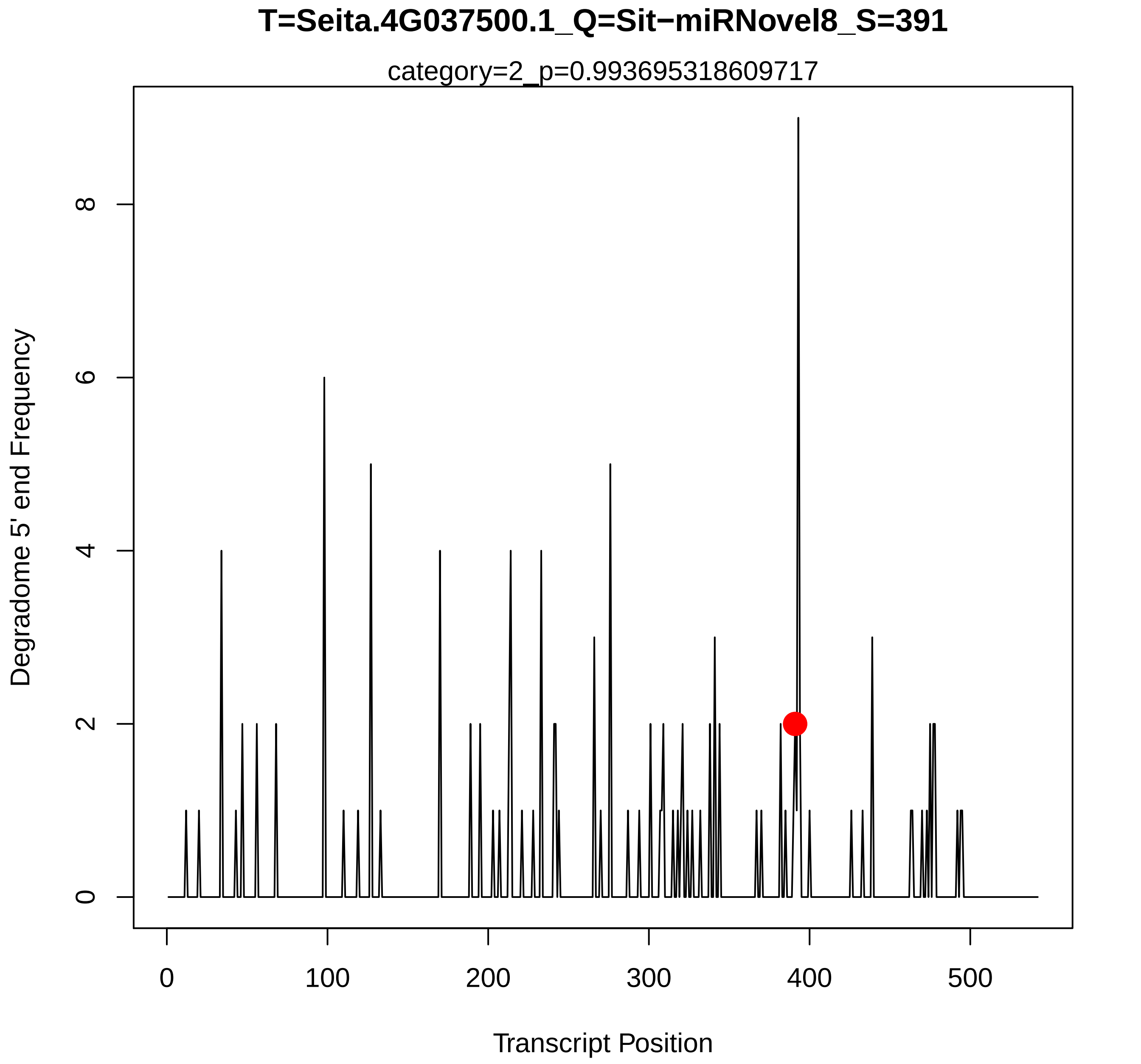

T=Seita.4G037500.1_Q=Sit−miRNovel8_S=391
category=2_p=0.993695318609717
Degradome 5' end Frequency
0
2
4
6
8
0
100
200
300
400
500
Transcript Position
